# Metabolic switch from glycogen to lipid in the liver maintains glucose homeostasis in neonatal mice

**DOI:** 10.1016/j.jlr.2023.100440

**Published:** 2023-10-11

**Authors:** Liangkui Li, Haoyu Zhou, Jinhui Wang, Jiaxin Li, Xuchao Lyu, Wenshan Wang, Chengting Luo, He Huang, Dawang Zhou, Xiaowei Chen, Li Xu, Peng Li

**Affiliations:** 1 State Key Laboratory of Membrane Biology and Tsinghua-Peking Center for Life Sciences, School of Life Sciences, Tsinghua University, Beijing, China; 2 Tianjian Laboratory of Advanced Biomedical Sciences, Zhengzhou University, Zhengzhou, China; 3 The Institute of Metabolism and Integrative Biology, Fudan University, Shanghai, China; 4 State Key Laboratory of Cellular Stress Biology, Innovation Center for Cell Signaling Network, School of Life Sciences, Xiamen University, Xiamen, Fujian, China; 5 College of Future Technology, Peking University, Beijing, China

**Keywords:** neonates, glycogen, lipids, gluconeogenesis, glucose homeostasis, biological sciences, Physiology

## Abstract

Neonates strive to acquire energy when the continuous transplacental nutrient supply ceases at birth, whereas milk consumption takes hours to start. Using murine models, we report the metabolic switches in the first days of life, with an unexpected discovery of glucose as the universal fuel essential for neonatal life. Blood glucose quickly drops as soon as birth, but immediately rebounds even before suckling and maintains stable afterward. Meanwhile, neonatal liver undergoes drastic metabolic changes, from extensive glycogenolysis before suckling to dramatically induced fatty acid oxidation (FAO) and gluconeogenesis after milk suckling. Unexpectedly, blocking hepatic glycogenolysis only caused a transient hypoglycemia before milk suckling without causing lethality. Limiting lipid supply in milk (low-fat milk, [LFM]) using *Cidea*^*−/−*^ mice, however, led to a chronic and severe hypoglycemia and consequently claimed neonatal lives. While fat replenishment rescued LFM-caused neonatal lethality, the rescue effects were abolished by blocking FAO or gluconeogenesis, pointing to a funneling of lipids and downstream metabolites into glucose as the essential fuel. Finally, glucose administration also rescued LFM-caused neonatal lethality, independent on FAO or gluconeogenesis. Therefore, our results show that the liver works as an energy conversion center to maintain blood glucose homeostasis in neonates, providing theoretical basis for managing infant hypoglycemia.

Nutrient availability is a strong selective pressure that shapes metabolic processes, allowing adaptation to the different energy sources. Mammals undergo an essential yet daunting metabolic switch from embryonic to neonate stages. In utero, the fetus is supplied with continuous transplacental transfer of nutrients from the maternal circulation, using glucose as the predominant energy source (1, 2). Immediately at birth, however, this continuous transplacental supply of nutrient ceases. For survival and proper development, the postneonates have to first withstand the unavailability to external nutrition, prior to switching to lipid-rich milk after several hours (3). Of note, maladaptation to the metabolic switch in neonates causes consistent or recrudescent hypoglycemia, a common pathological mechanism (3, 4) that could result in neural damage and developmental retardation in infants (5, 6, 7). Despite the essentiality of the swift metabolic adaptation, the underlining regulatory mechanisms that maintain glucose supply amid the drastic nutritional switch in neonates still remains elusive.

Milk lactation is a defining characterization of mammals and has been considered as one of the important features of evolution (8, 9). Fat, protein, and lactose constitute the common fuel sources, with fat accounting for more than half of the calories (10, 11, 12). Unlike the relative constant protein and lactose contents in milk among different species, the fat contents in milk could vary from 3.8% in humans to 18.0% in mice (13, 14). Moreover, reduction in fat contents of mouse milk results in growth retardation to gradually increased lethality in the neonates (15, 16, 17, 18, 19). Accordingly, human genetic defects in the transport or oxidation of fatty acids are associated with hepatic encephalopathy, in which the patients (typically children) develop severe hypoglycemia and sudden onset of liver failure (20, 21, 22). While these studies strongly implicate lipid as a pivotal fuel source in neonates, the exact role of lipid utilization in supporting the development and growth of the neonates remains elusive.

Though not as energy-rich as lipids, glucose plays a pivotal and versatile role in fuel supply, capable of producing ATP in both anaerobic and aerobic conditions. Moreover, certain tissues including the brain almost entirely rely on glucose as fuel molecules. Besides dietary uptake, glucose can be provided by mobilization of glycogen stores or gluconeogenesis in times of fasting or starvation (23, 24). Moreover, while hepatic gluconeogenesis is typically suppressed during feeding in healthy adults, unsuppressed gluconeogenesis in postprandial states accounts in part for the hyperglycemic phenotypes in diabetic patients.

The present study focused on the metabolic changes in neonates and found that neonatal mouse liver serving as an energy conversion center, senses environmental nutrients and contributes to maintaining glucose homeostasis. Glycogen and lipids are two main forms of energy source in vivo. We used two animal models, which blocks hepatic glycogen mobilization or limit lipid supply from milk, respectively, to demonstrate how neonates relay different energy substrates to maintain constant glucose levels through regulating differently metabolic pathways.

## Materials and methods

### Generation of adipocyte-specific KO mice and animal maintenance

Generation of *Pygl* liver-specific KO mice was described in previous study (25), which was gifted for the study. Briefly, the *Pygl* floxed allele was constructed by a LoxP sites flanking exon 4 targeting vector. The isogenic crosses between *Pygl*^*flox/flox*^ and *Pygl*^*flox/flox*^ with albumin-Cre, which generated newborns *Pygl*^*flox/flox*^ (*Pygl*^*+/+*^, control group) and newborns *Pygl*^*flox/flox*^ with albumin-Cre (*Pygl*^*−/−*^, experimental group). The primer sequences used to determine albumin-Cre were 5′- GAAGCAGAAGCTTAGGAAGATGG-3′ and 5′- TTGGCCCCTTACCATAACTG-3′. Generation of *cidea* deficiency mouse was generated in our previous study (26). Briefly, a replacement targeting construct was prepared in which the Cidea sequence between *Eco*RI and *Pst*I, including half of the second exon, was removed and replaced with *neo*^r^. The genotyping primer sequences used to confirm *cidea* deficiency mouse were 5′-GCCCCAGGCCTGGACTCTGAGCTAG-3′ and 5′-GGCACAGAACCAAAACCCCGAAGTG-3′. Mouse experiments were performed in the animal facility of Tsinghua University (Beijing, China). All animal experiments were approved by the Institutional Animal Care and Use Committee of Tsinghua University. Mice were housed in a temperature-controlled environment (22°C, 12-h light/dark cycle) with free access to water and standard rodent chow diet.

### Milk collection and analysis

Milk was collected from lactating female mice as described (27). Briefly, female mice were separated from their pups at day 1 of lactation for 3–4 h and injected with 10 units of oxytocin (Sigma-Aldrich). At 10 min after the injection, milk was manually collected from the fourth pair of mammary glands for further analysis.

### Lipidomics analysis of milk lipids

Lipids were extracted from milk as described (28, 29). Liquid chromatography-tandem mass spectrometry (LC-MS/MS) method for lipid analysis have been previously described (30, 31). Briefly, reverse phase chromatography was selected for LC separation using Cortecs C18 column (2.1 × 100 mm, Waters). Mobile phase A was made by mixing 400 ml of HPLC-grade water containing 0.77 g of ammonium acetate with 600 ml of HPLC-grade acetonitrile (pH ∼ 7). Mobile phase B contained 10% acetonitrile and 90% isopropanol (v/v). Data were acquired using QExactive orbitrap mass spectrometer (Thermo Fisher Scientific, CA) coupled with UHPLC system Ultimate 3000 (Thermo Fisher Scientific, CA).

### Metabolomics analysis of milk and tissues

Extraction of metabolites from milk and liver have been previously described (32). Briefly, adding 500 μl of 80% (vol/vol) HPLC-grade methanol (cooled to −80°C) to frozen tissue pieces (nearly 40 mg liver), which were then grinded for 1–2 min on the dry ice. After centrifugation at 18,000 *g* for 10 min, the supernatant of the tissue homogenates or milk were transferred to a new eppendorf tube, and then stored at −80°C. Adding 400 μl of 80% (vol/vol) methanol (−80°C) to the precipitate, which were then vortexed for 1 min at 4–8°C. The supernatant was transferred and combined from both the extractions. The combined supernatants were dried to a pellet using no heat. The metabolites were then further analyzed by LC-MS/MS (33).

### Proteomics analysis of milk

Thirty microliters of mice milk were lysed using urea (8 M), and the whole extracts were centrifuged at 14,000 *g* for 20 min at 4°C. Protein concentrations were determined with the bicinchoninic acid method. Equal amounts of protein were reduced with 5 mM dithiothreitol and alkylated with 13 mM iodoacetamide. In-solution digestion was then carried out with sequencing-grade trypsin at 37°C overnight. The liquid chromatography tandem mass spectrometry (LC-MS/MS) analysis was used in this study (34).

### Serum biochemical analysis

Blood glucose concentrations were measured through tail veil bleeding with glucose analyzer (GM9, Analox Instruments Ltd, UK). The concentrations of serum triacylglycerides (TAGs) and nonesteriﬁed fatty acids (NEFAs) were measured using TAG reagent (Sigma-Aldrich, T2449) and free glycerol reagent (Sigma-Aldrich, F6248), and a Lab Assay NEFA kit (Wako Pure Chemical Industries, Japan, 294-63601), respectively. Serum ketone body concentrations and serum alanine were determined by Ketone Body Assay (Sigma-Aldrich, MAK134) and Alanine assay kit (Sigma-Aldrich, MAK001), respectively. Milk lactose was measured by Lactose Assay Kit (Sigma-Aldrich, MAK017). Pyruvate carboxylase activity was determined by Pyruvate Carboxylase Activity Assay Kit (Boxbio, AKSU070M). Brain ATP levels were determined by ATP Assay Kit (Beyotime, S0026).

### Gene expression by quantitative PCR

In milk, microRNA was extracted using microRNA kit (SNC50-1KT) accordance with the manufacturer’s instructions. cDNA synthesis was performed using *TransScript*® Green miRNA Two-Step qRT-PCR SuperMix (Sigma-Aldrich, AQ202-01). In tissues, total RNA was isolated using TRIzol (Thermo Fisher Scientific) in accordance with the manufacturer’s instructions. First strand cDNA synthesis was conducted with RevertAid First Strand cDNA Synthesis Kit (Thermo Fisher Scientific). Real-time reverse transcriptase-PCR detection of gene expression levels were analyzed using the Power SYBR Green PCR Master Mix (Applied Biosystems) on an ABI 7500 (Applied Biosystems) with reaction volumes of 20 ml. Beta-actin gene was used as the reference gene. The primer sequences used are listed in supplemental Table S1.

### Protein expression by immunoblotting analyses

The frozen tissues were homogenized in a lysis buffer (20 mM Tris-HCl, 150 mM NaCl, 1 mM EDTA, 1 mM EGTA, 1% Triton-X100, and protease inhibitor, pH 7.4) and then centrifuged for 20 min at 10,000 *g* to discard cell debris. The total protein concentrations were determined using a Bio-Rad kit. The proteins were subjected to Western blot analysis with the desired antibodies. Antibodies against carnitine palmitoyltransferase 1A (CPT1A) (ab234111), HMGCS2 (ab137043), FAS (ab128870), HMGCR (ab174830), liver glycogen phosphorylase (PYGL) (ab198268) and PYGL (phospho S15, ab227043) were obtained from Abcam. Antibodies against COX4 (A21348) was obtained from Invitrogen. Antibody against phosphoenolpyruvate carboxykinase 1 (PCK1) (A2036) and GCK (A6293) was purchased from ABclonal. Antibodies against AMPKα (2532), phospho-AMPKα (2535), CREB (9197), Phospho-CREB (9198), and SCD1 (2438) were purchased from Cell Signaling Technology. Antibody against G6PC (22169-1-AP) was purchased from Proteintech. Antibody against TUBULIN (T0198) was obtained from Sigma-Aldrich.

### Measurement of tissue lipid content

Isolation and measurement of total lipid from mouse tissues have been previously described with minor modifications (28, 29). Briefly, tissues (nearly 30 mg liver) were homogenized in PBS buffer with protease inhibitors. A chloroform/methanol (2:1) solution was rapidly added to the homogenate and the samples were vortexed. After centrifugation at 1,000 *g* for 5 min, the upper organic phase was collected and dried under nitrogen gas. Lipids were resuspended in chloroform containing 1% Triton X-100 (1 ml) and then dried with nitrogen gas. Lipids were finally resuspended in water (200 μl). Isolated lipids were diluted for detection accordingly. TAG and free fatty acids levels were measured using TAG reagent (Sigma-Aldrich) and free glycerol reagent (Sigma-Aldrich), and a Lab Assay NEFA kit (Wako Pure Chemical Industries, Japan), respectively.

### Determination of glucose and glycogen level

The glucose and glycogen levels of mouse tissues were measured as previously described (35). Briefly, 30 mg of the liver or 50 mg of the brain tissue was homogenized in 1 ml pre-cooled 6% perchloric acid (Sigma-Aldrich), followed by centrifugation at 14,000 *g* at 4°C for 10 min. The supernatant (S1) were neutralized with KOH and centrifuged. The supernatant (S2) was removed, and 20 μl of S2 was added to 100 μl of 1 mg/ml amyloglucosidase (Sigma-Aldrich, A7420) in 0.2 M acetate buffer (pH 4.8) for digestion at 50°C for 2 h. After the digestion, the solution (S3) was taken for glucose measurement by a glucose assay kit (Sigma-Aldrich, GAGO20). The glycogen level was calculated by subtracting the basal glucose level in S2 from that of S3.

### Fat emulsion feeding and glucose injection

From 6 h after birth, the pups lactated by *Cidea*^−/−^ females (low-fat milk, LFM) were orally administrated with 25 μl/g (body weight) of 20% (w/w) fat emulsion, which contains soybean oil, 20% (w/v); lecithin, 1.2% (w/v); and glycerol, 2.2% (w/v) that was orally supplemented every 6 h. Or the pups were injected with 50 μl/g of 5% glucose in PBS every 8 h. Pent-4-enoate (fatty acid oxidation [FAO] inhibitor, Selleck, 2 mg/g body weight) or 3-MPA (gluconeogenesis inhibitor, MedChemExpress, 100 μg/g body weight) was injected every 8 h. After the treatments, the pups were immediately returned to the cages and stayed with their moms.

### Histological analysis

Livers from mice were excised and ﬁxed in 10% formalin buffer, dehydrated, embedded in parafﬁn blocks and sectioned at 5 μm. The sections were then stained with haematoxylin and eosin. Livers from mice were excised and ﬁxed in 10% formalin buffer, and the fixed specimens were sectioned and stained with Oil Red O. For electron microscope (EM) analysis, livers were fixed in 2.5% glutaraldehyde buffer and studied at Center of Biomedical Analysis, Tsinghua University, China.

### Statistics

All statistical analyses were performed in GraphPad Prism, Version 5 (https://www.graphpad.com, GraphPad Software). Data are presented as mean values ± s.e.m. Data were analyzed using a two-tailed Student’s *t* test. ∗*P* <0.05, ∗∗*P* <0.01, ∗∗∗*P* <0.001.

## Results

### Fuel switch and metabolic adaptation in neonatal liver

**Fig. 1:**
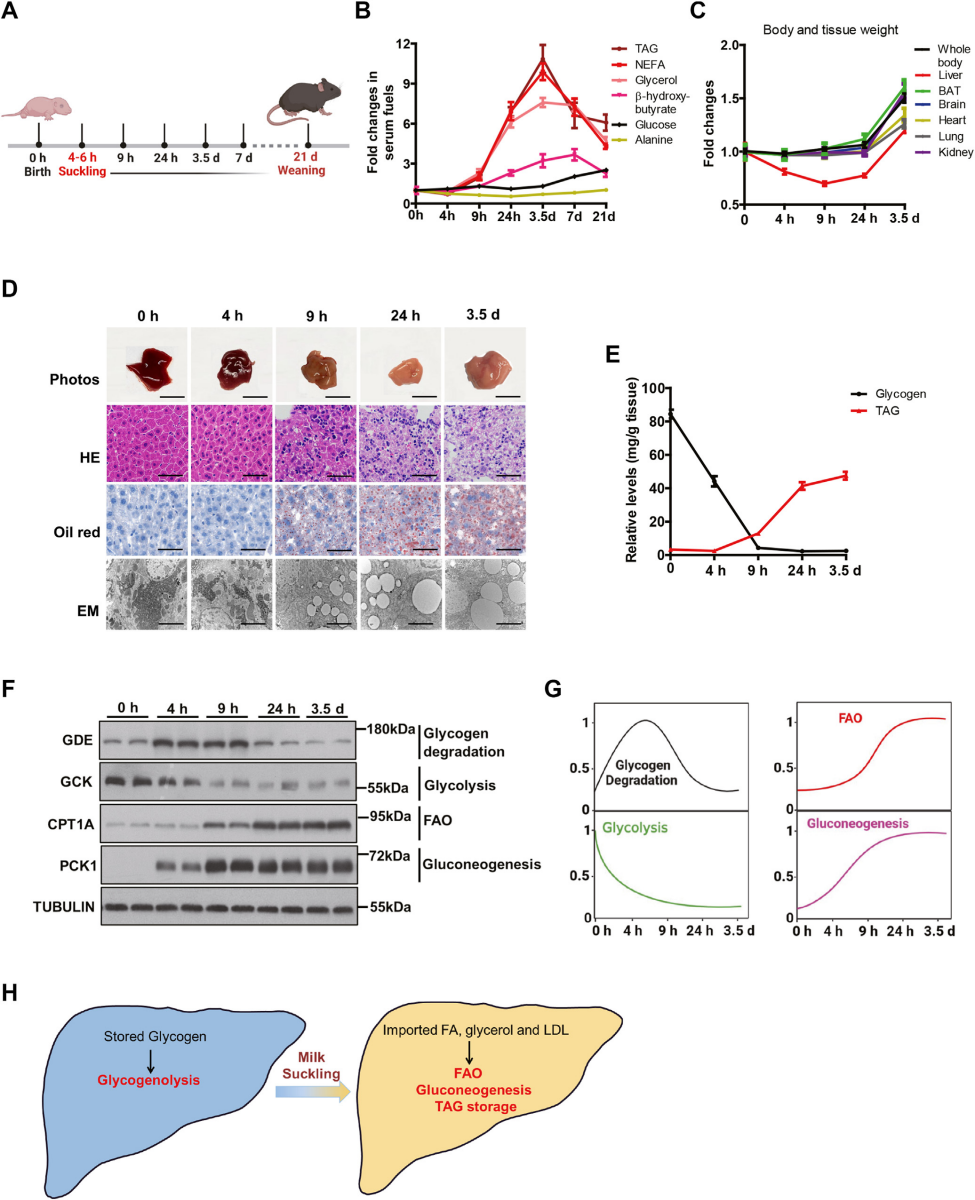
Fuel switch and metabolic adaptation in neonatal liver. A: Schematic of sampling time according to neonatal physiology. B: Temporal change folds of different fuels in serum. The parameter levels at birth were set to one, respectively (n = 6 per group). C: Temporal change of body and tissue weights. The weights at birth were set to one, respectively (n = 6 per group). D: Liver photos and liver slice stained by HE, oil red O staining for lipids, and EM (n = 3). Scale bar represented 0.5 cm in the liver photos. Scale bar represented 50 μm in the upper and middle rows of images, and 2 μm in the bottom row of images. E: Relative levels of glycogen and TAG in liver (n = 6). F: Western blotting showing the protein levels of GDE, GCK, CPT1A, and PCK1 in the neonatal liver at 0, 4, 9, and 24 h and 3.5 days after birth (n = 2). G: Temporal changes in the activity of the respective metabolic pathway alternations in livers during 3.5 days after birth. For a given protein, its highest intensity is set as 1 and relative levels are compared with the highest level according to (F). H: A working model of metabolic switches from the glycogenolytic to the FAO/gluconeogenic liver in neonates. Data are presented as mean ± s.e.m. Data were analyzed using a two-tailed Student’s *t* test. ∗*P* < 0.05, ∗∗*P* < 0.01, ∗∗∗*P* < 0.001. CPT1A, carnitine palmitoyltransferase 1A; EM, electron microscope; FAO, fatty acid oxidation; GDE, glycogen debranching enzyme; PCK1, phosphoenolpyruvate carboxykinase 1; TAG, triacylglyceride.

Murine neonates start milk suckling around 4–6 h after birth, until their weaning around 21 days. We thus set up the sampling time according to the neonatal physiology, in order to characterize the metabolic changes in the beginning of the new life (Fig. 1A). To this end, different fuel molecules in serum were temporally monitored, including the lipid-related ones (TAG, NEFA, glycerol, and β-hydroxybutyrate), glucose, and the glucogenic amino acid alanine, all of which are critical for the survival and growth of the pups (36) (Fig. 1B). Interestingly, the levels of TAG, NEFA, and glycerol increased sharply after 9 h postnatal and reached a peak (7.5–11.0 fold) at 3.5-days, followed by a rapid decrease during 3.5–7.0 days, and a slower decrease until 21-days, though the final concentrations were still 4–6 fold higher than those at birth (Fig. 1B and supplemental Fig. S1A–C). These data indicated an activation of lipid metabolism in neonatal mice within the first week. β-Hydroxybutyrate and glucose can cross the blood-brain barrier and therefore are used by the brain as fuels. Interestingly, β-hydroxybutyrate reached a peak with ∼4-fold over basal at 7-days, with a slow decline until 21 days (2.3-fold, Fig. 1B and supplemental Fig. S1D). Serum glucose was maintained within a narrow range at first 3.5-days (3.6–4.8 mmol/L), followed by a slow increase until 9.1 mmol/L at 21-days under a random feeding condition (Fig. 1B and supplemental Fig. S1E). Other serum fuels changed dramatically during 21 days after birth, while serum alanine level always remained at a very stable level during the 21 days (Fig. 1B and supplemental Fig. S1F). These data revealed a drastic elevation of lipid utilization that occurs within the first 24 h of life and persists afterward, plus a steady increase of circulating glucose supply.

Next, we monitored the changes in body weight and tissue weight, which reflect the growth and development of the pups. As expected, body weight and weights of most tissues remained steady within the first 24 h after birth and started to increase afterward (Fig. 1C). In sharp contrast, liver weight significantly dropped during postnatal 24 h and only returned to the initial weight after 3.5 days (Fig. 1C), reflecting a drastic metabolic change within the tissue.

The outstanding drop in neonatal liver weight intrigued us to examine morphological changes at different scales. The neonatal liver appeared reddish at birth and gradually turned pale after 9 h post birth. Haematoxylin and eosin and Oil Red staining both revealed an induction of lipid droplets at 9 h post birth. Ultrastructural analysis by EM confirmed the above induction of lipid droplets and further revealed a depletion of glycogen particles within the first 9 h after birth (Fig. 1D). Consistent with the morphological analysis, biochemical isolation also revealed that a large amount of glycogen was accumulated within fetal liver at birth, but dramatically decreased at 4 h after birth, and became almost depleted at 9 h after birth (Fig. 1E). By contrast, TAG levels were low at birth, but quickly increased in the liver after suckling (at 4 h after birth) and continued to accumulate in the neonatal liver (Fig. 1E). Taken together, these data further supported a substantial change in hepatic metabolism during 3.5-days postnatal.

We further surveyed the temporal changes of metabolic pathways for the respective fuels at the molecular level. Glycogen debranching enzyme (GDE) is required for efficient glycogen degradation and is regulated by ubiquitination (37). GDE level significantly increased at 4 and 9 h after birth, but dramatically decreased at postnatal 24 h and 3.5 days (Fig. 1F). PYGL is the first enzyme for hepatic glycogen degradation, whose activity is regulated by glucagon-induced phosphorylation (23). PYGL was drastically phosphorylated at 4 h and its phosphorylation status was maintained at a similar level after 4 h (supplemental Fig. S1G). The expression and activation of these enzymes in glycogen degradation was consistent with the levels of hepatic glycogen, suggesting an active glycogen breakdown after birth until postnatal 9 h. Of note, gluconeogenic enzymes, such as *Pck1* and glucose-6-phosphatase (*G6pc*), were sharply induced at 4 h and continuously maintained at a high level later (Fig. 1F and supplemental Fig. S1H), despite the abundant caloric supply of milk. In sharp contrast, glycolysis (*Gck*, P*fkl*, and *Pklr*) was blocked after birth (Fig. 1F and supplemental Fig. S1I). By contrast, the expression levels of key genes in fatty acid transport (*Fatp2* and *Fatp5*) and oxidation (*Cpt1a*) were sharply induced at 9 h and continued to increase (supplemental Fig. S1J). Similar increase was observed with CPT1A protein, together coinciding with the onset of lipid-rich milk feeding (Fig. 1). Ketogenesis (*Hmgcs2*, *Hmgcl*, and *Bdh1*) was also induced after birth to a less degree compared to that of FAO and gluconeogenesis (supplemental Fig. S1G, K). In addition, de novo lipogenesis (*Srebp1c*, *Acc1*, *Fasn*, *Elvol6*, and *Scd1*) and cholesterol synthesis (*Hmgcs1*, *Hmgcr*, *Fdft1*, and *Lss*) were also blocked after birth, indicating an external source of hepatic accumulation observed above, consistent with milk consumption (supplemental Fig. S1L, M).

Taken together, the above biochemical and molecular analysis led to a model of metabolic switch from glycogenolytic to FAO/gluconeogenic states in neonatal liver (Fig. 1G), characterized by a drastic shift from glycogen to lipid metabolism, accompanied by enhanced gluconeogenesis and fatty acid oxidation (Fig. 1H).

### Hepatic glycogen maintains blood glucose homeostasis in the first hours after birth

The activation of hepatic glycogen degradation after birth intrigued us to determine the role of this fuel supply to neonatal physiology. To this end, we employed animals harboring liver-specific deletion of *Pygl* (*Pygl*^*−/−*^, *Pygl* liver-specific knock out (LKO)), as deficiency of the key enzyme in glycogenolysis abolishes glycogen utilization (supplemental Fig. S2A) (25). The deficiency of *Pygl* had no detectable effects on neonatal survival and body weight compared with that of *Pygl*^*+/+*^ neonates throughout postnatal development (supplemental Fig. S2B, C), consistent with the previous report related to *Pygl*^*−/−*^ adult mice (23).

**Fig. 2:**
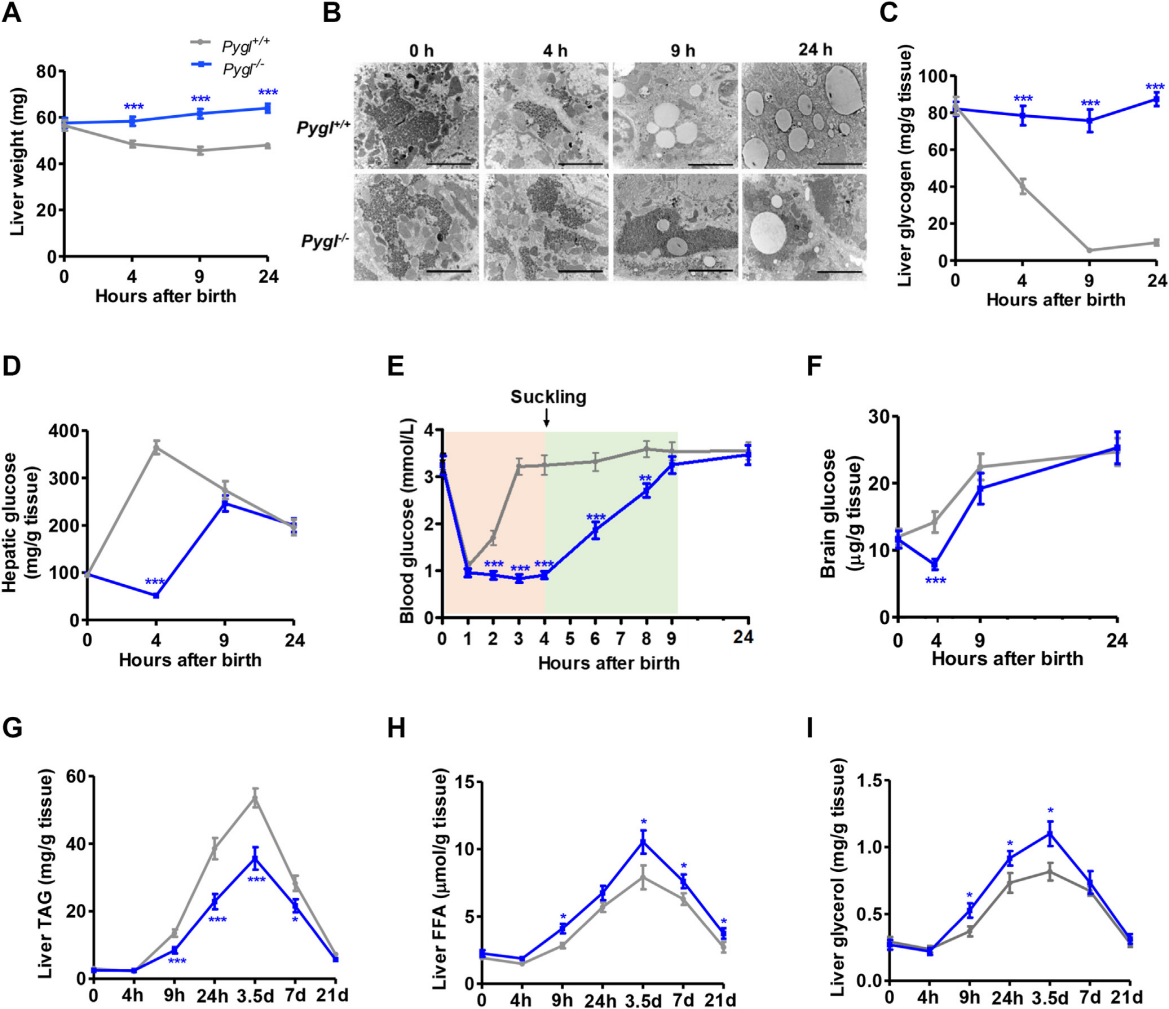
Hepatic glycogen maintains blood glucose homeostasis in the first hours after birth. A: Liver weight of *Pygl*^*+/+*^ and *Pygl*^*−/−*^ neonates at the indicated time (n = 8 per group). B: Representative liver EM. Scale bar represented 2 μm in the images (n = 3). C: Hepatic glycogen level (n = 6). D: Hepatic glucose level (n = 6). E: Blood glucose level (n = 10). F: Brain glucose level (n = 6). G–I: Hepatic TAG (G), FFA (H), and glycerol (I) level (n = 6). Data are presented as mean ± s.e.m. Data were analyzed using a two-tailed Student’s *t* test. ∗*P* < 0.05, ∗∗*P* < 0.01, ∗∗∗*P* < 0.001. EM, electron microscope; TAG, triacylglyceride.

Of note, the reduction of neonatal liver weight in WT mice was absent in *Pygl*^*−/−*^ liver (Fig. 2A). Accordingly, in contrast to the depleted hepatic glycogen after postnatal 9 h in controls, *Pygl*^*−/−*^ liver maintained a substantial amount of glycogen, as visualized by EM and biochemical analysis (Fig. 2B, C). Given the role of hepatic glycogen mobilization in maintaining plasma glucose concentration in response to fasting, we next examined glucose levels in the liver, blood, and brain from *Pygl*^*+/+*^ and *Pygl*^*−/−*^ neonates. In control *Pygl*^*+/+*^ neonates, hepatic glucose rapidly rose to a peak at 4 h (Fig. 2D, gray line). By contrast, *Pygl*^*−/−*^ neonates experienced a reduction in hepatic glucose after birth, and their hepatic glucose levels only started to increase after 4 h to match to those of *Pygl*^*+/+*^ counterparts at 9 h. Coincided with the hepatic glucose levels, blood glucose levels in *Pygl*^*−/−*^ neonates remained diminished in the first 9 h after birth, yet were able to quickly rebounded to the initial birth levels in the controls (Fig. 2E). Intriguingly, the elevation of blood glucose appeared when the neonates started milk suckling at 4–6 h postnatal.

Postnatal brain exclusively relies on glucose, supplied via the blood stream, as the energy fuel. Indeed, the depletion of hepatic and circulating glucose in *Pygl* LKO consequently lowered glucose concentration in brain after birth. However, the diminished brain glucose level in *Pygl* LKO became normalized as controls at 9 h (Fig. 2F), also coinciding with milk suckling. Unlike glucose, other fuels including TAG, FFA, glycerol, and β-hydroxybutyrate exhibited no detectable differences between *Pygl*^*+/+*^ and *Pygl*^*−/−*^ neonates within 24 h (supplemental Table S2). These data indicated that blockade of hepatic glycogen mobilization and glucose production, via Pygl inactivation, caused neonatal hypoglycemia until milk suckling.

We also observed that *Pygl*^*−/−*^ liver contained less TAG than WT liver throughout the first 21 days after birth (Fig. 2G). Meanwhile, hepatic levels of FFA and glycerol, products of TAG mobilization, were always higher in *Pygl*^*−/−*^ mice than those from WT mice (Fig. 2H, I). Unlike the alterations in glycogen and lipid metabolism, however, loss of hepatic *Pygl* did not alter the protein expression of key enzymes involved in glycolysis (G6PC), gluconeogenesis (PCK1), FAO (CPT1A), or PKA signaling (phosphorylated CREB) (supplemental Fig. S2D). Taken together, these data demonstrate that, hepatic glycogenolysis is responsible for maintaining glucose hemostasis within the first 9 h after birth, whereas defective glycogen mobilization in the liver might lead to a compensatory increase on hepatic lipid utilization.

### Milk lipids sustain blood glucose homeostasis and neonatal survival

The upregulation of hepatic lipid utilization, plus unexpected replenishment of defective glucose supply at the time of milk suckling, intrigued us to examine milk lipids in maintaining glucose supply to support the neonatal life. To selectively deplete lipid supply from milk, we used *Cidea* deficiency mouse model, as milk secreted from *Cidea*^*−/−*^ females contains much less lipids (15). Consistent with the previous report, TAG was reduced by ∼70% in milk collected from female *Cidea*^*−/−*^ mice compared with the one from *Cidea*^*+/+*^ mice (40 vs. 140 mg/ml) (supplemental Fig. S3A). Lipidomics analysis confirmed the drastically different lipid profiles from mice with *Cidea*^*+/+*^ or *Cidea*^*−/−*^ genotypes (supplemental Fig. S3B). No significant difference was observed in milk proteins, metabolites, lactose, and microRNA between *Cidea*^*+/+*^ and *Cidea*^*−/−*^ milk based on titer detection, proteomics, and metabolomics, respectively (supplemental Fig. S3B–F). Hence, these data indicated that lipids are selectively depleted in milk produced by *Cidea*^*−/−*^ females, setting up the basis for us to determine the contribution of this major fuel source in neonatal life.

**Fig. 3:**
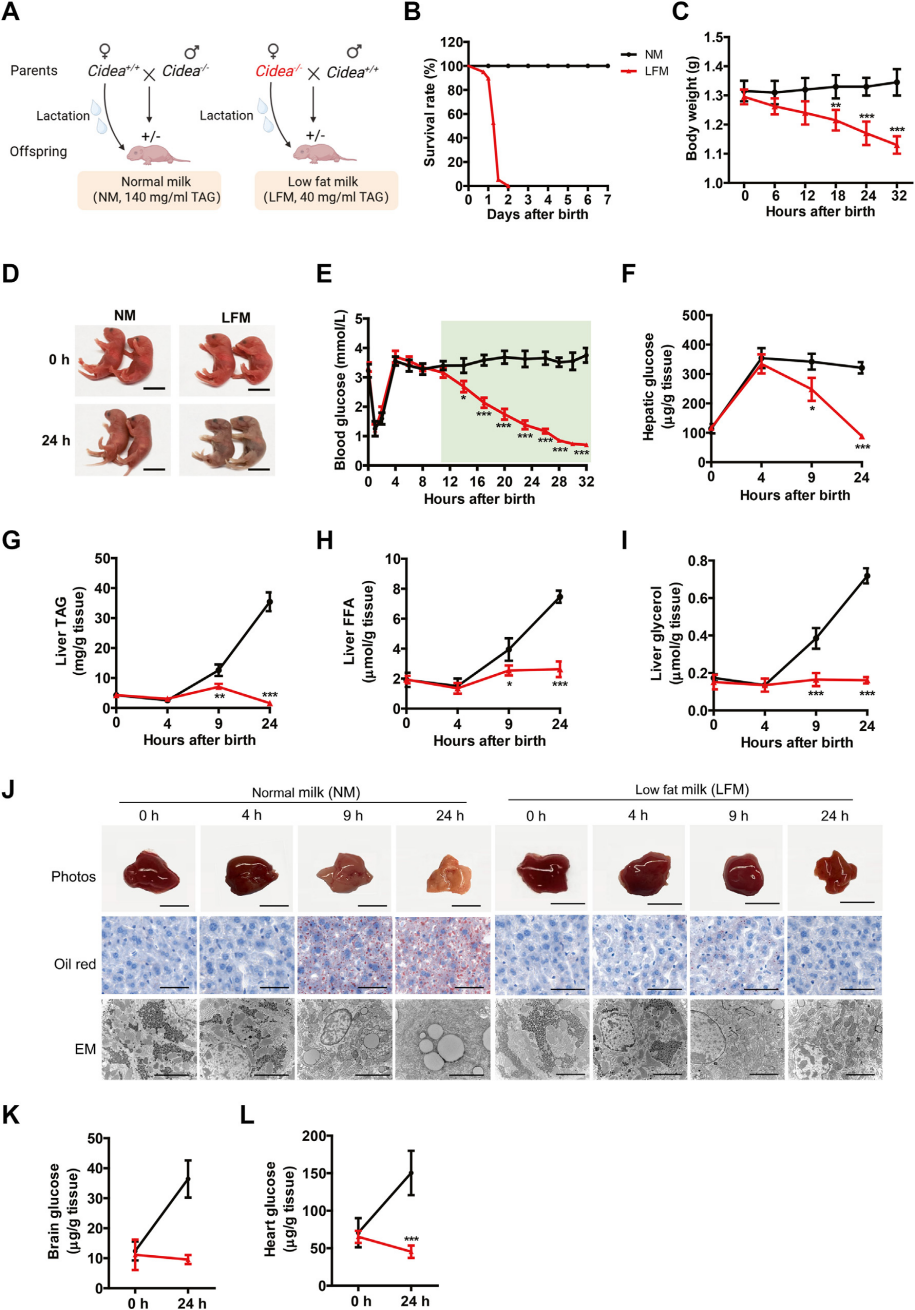
Milk lipids sustain blood glucose homeostasis for neonatal survival. A: Isogenic mating obtain offsprings with the same genotype, but lactated differently. The neonates were lactated by their own mother, namely by *Cidea*^*+/+*^ (normal milk, NM) and *Cidea*^*−/−*^ (low-fat milk, LFM) females for next experiments. B: Survival rates of neonatal mice suckling NM or LFM (n = 20 per group). C: Body weights of the indicated neonates during postneonatal 32 h (n = 6). D: Representative images of neonates lactated by *Cidea*^*+/+*^ and *Cidea*^*−/−*^ females at 0 (at birth) and 24 h after birth (n = 2). Scale bar represented 1 cm. E: Blood glucose levels in the neonates (n = 10). F–I: Levels of hepatic glucose (F), TAG (G), FFA (H), and glycerol (I) of neonates lactated by NM or LFM at 0, 4, 9, and 24 h after birth (n = 6). J: Liver photos and morphology of neonatal mice at 0, 4, 9, and 24 h after birth (n = 3). Oil Red O staining, and EM were performed. Scale bar represented 0.5 cm in the liver photos. Scale bar represented 50 μm in the upper row of images and 2 μm in the bottom row of images. K, L: Glucose levels of the brain (K) and heart (L) from the neonates lactated by NM and LFM at 24 h after birth, respectively (n = 6). Data are presented as mean ± s.e.m. Data were analyzed using a two-tailed Student’s *t* test. ∗*P* < 0.05, ∗∗*P* < 0.01, ∗∗∗*P* < 0.001. EM, electron microscope; TAG, triacylglyceride.

Namely, *Cidea*^*+/−*^ pups produced by *Cidea*^*+/+*^ females would receive normal milk (NM), whereas *Cidea*^*+/−*^ pups produced by *Cidea*^*−/−*^ females would instead receive the LFM. Of note, the pups fed with LFM died within 32 h after birth (Fig. 3B), with decreased body weights compared to the isogenic ones receive NM (Fig. 3C). The dying pups started to show skin cyanosis for nearly 15–30 min (Fig. 3D), but with normal lung morphology and no apparent breathing difficulty (supplemental Fig. S3G).

Of note, while similar levels of blood glucose were observed in both groups before 8 h after birth, blood glucose continued to decline in the LFM-fed pups and reached a level of severe hypoglycemic prior to a sudden death at 32 h (Fig. 3E). Moreover, levels of hepatic glucose were also depleted in LFM-fed pups compared to pups receiving NM (Fig. 3F), as depleted were the levels of hepatic TAG, FFA, and glycerol (Fig. 3G–I). Consistent with the biochemical analysis, LFM-fed mice exhibited drastically different appearance from the controls receiving NM (Fig. 3J), reflecting the absence of lipid storage. Finally, LFM-fed pups also contained less glucose in brain and heart than the controls receiving NM (Fig. 3K, L), further confirming the hypoglycemia caused by the limited lipids in milk supply.

### Metabolomics showing milk fat utilization is one of the essential cofactors of gluconeogenesis

**Fig. 4:**
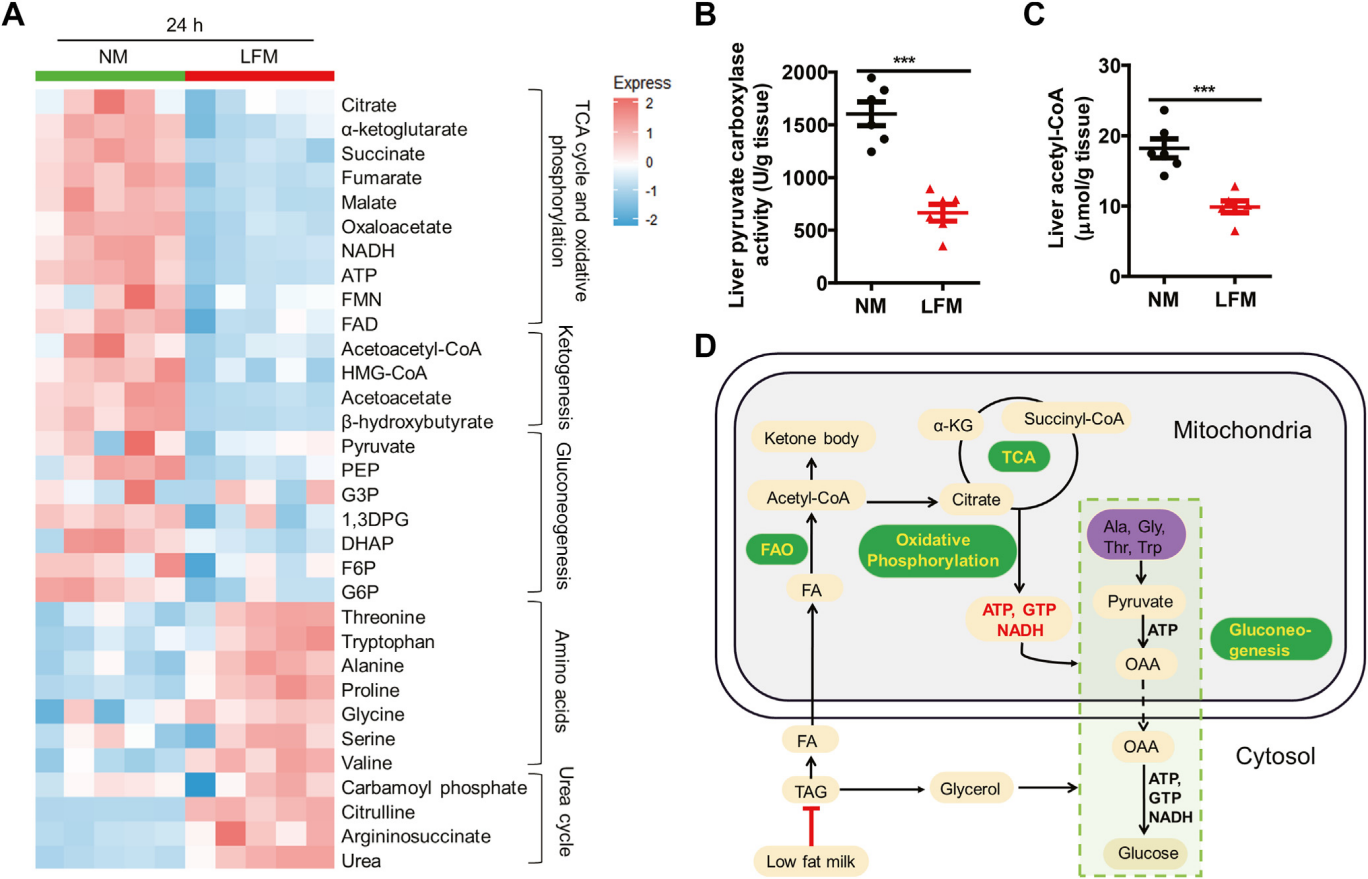
Metabolomics milk showing fat utilization is the essential cofactors of gluconeogenesis. A: Metabolomics heatmap of liver at postnatal 24 h (n = 5). The liver samples were collected from the mouse neonates as illustrated in Fig. 3. B, C: Hepatic levels of liver pyruvate carboxylase activity (B) and acetyl-CoA (C) of mice lactated by normal milk (NM) or low-fat milk (LFM) at postnatal 24 h (n = 6). D: The schematic summary. Neonatal liver actively produce glucose through FAO and gluconeogenesis. Low milk lipid significantly reduced imported FFA and glycerol into the liver, consequently blocking FAO and gluconeogenesis. Data are presented as mean ± s.e.m. Data were analyzed using a two-tailed Student’s *t* test. ∗*P* < 0.05, ∗∗*P* < 0.01, ∗∗∗*P* < 0.001. FAO, fatty acid oxidation.

To understand the molecular mechanism underlining the channeling of lipids into glucose, we performed untargeted metabolomics on NM-fed and LFM-fed pups, respectively. The levels of the metabolites in processes including the tricarboxylic acid cycle and oxidative phosphorylation, ketogenesis, and glycolysis/gluconeogenesis pathways were significantly reduced in LFM-fed pups (Fig. 4A), accompanied by an accumulation of glucogenic amino acids and the metabolites produced in the urea cycle (Fig. 4A). The data suggested reduced gluconeogenesis in pups receiving LFM. Consistent with this notion, the activity of pyruvate carboxylase (PC) was significantly blunted in the liver of LFM-fed pups compared to the controls (Fig. 4B). Along the same line, levels of the allosteric activator of PC, acetyl-CoA, were also diminished in livers of LFM-fed pups (Fig. 4C), as were energy molecules required for gluconeogenesis, including NADH and ATP (Fig. 4A). Hence, these molecular data support that reduced fat utilization limits glucose supply due to the lack of essential cofactors of gluconeogenesis (Fig. 4D).

### Dietary fat supports neonatal survival via FAO and gluconeogenesis

**Fig. 5:**
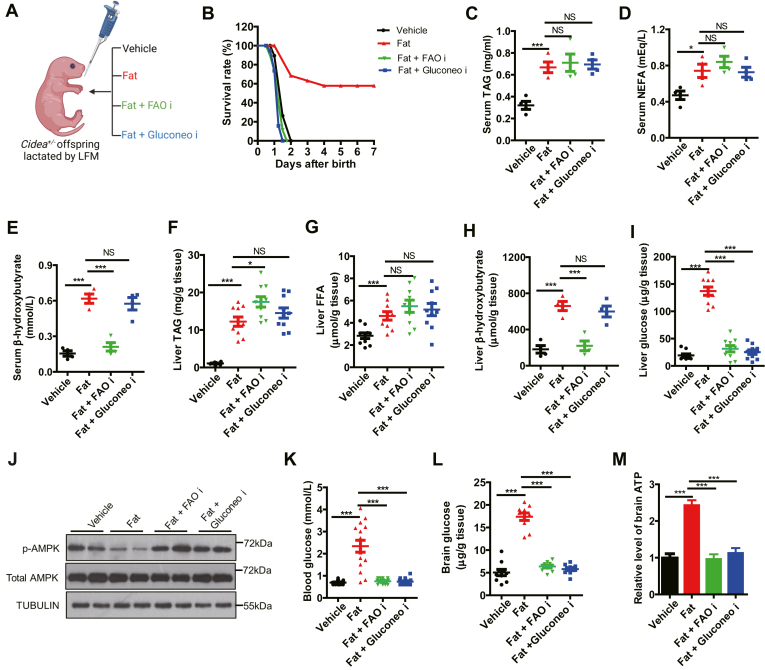
Dietary fat supports neonatal survival via FAO and gluconeogenesis. A: Schematic of the rescue experiments for (B–M). From 6 h after birth, the *Cidea*^*+/−*^pups lactated by *Cidea*^*−/−*^ females (low-fat milk, LFM) were orally administrated as four treatments: saline buffer as “vehicle”, fat emulsion supplementation as “Fat”, fat emulsion plus pent-4-enoate (FAO inhibitor) as “Fat + FAO i”, and fat emulsion plus 3-MPA (gluconeogenesis inhibitor) as “Fat + Gluconeo i”. B: The survival rates (n=15). C–E: Serum levels of TAG (C), NEFA (D), and β-hydroxybutyrate (E) (n = 4). F–I: Hepatic levels of TAG (F), FFA (G), β-hydroxybutyrate (H), and glucose (I) (n = 10). J: Protein level in livers by Western blotting (n = 2). K–M: The levels of blood glucose (K), brain glucose (I), and brain ATP (M) (n = 10). Data are presented as mean±s.e.m. Data were analyzed using a two-tailed Student’s *t* test. ∗*P* < 0.05, ∗∗*P* < 0.01, ∗∗∗*P* < 0.001. FAO, fatty acid oxidation; NEFA, nonesteriﬁed fatty acids; TAG, triacylglyceride.

The substantial glucose reduction with limited lipid supply raised the possibility that lipid consumption may fuel glucose production for neonatal survival. To test this hypothesis, we designed a set of rescue experiments based on the lethality caused by LFM (Fig. 5A). Nearly 60% of LFM-fed pups were rescued by fat readministration (*P* < 0.001). The rescue effects were blocked by FAO inhibitor that would prevent the utilization of the readministered lipids. Unexpectedly, the addition of gluconeogenesis inhibitor also completely blocked the rescue effects of lipid readministration (Fig. 5B). The data thus indicated that not only the energy supply of lipids but the channeling of lipids to glucose was required for neonatal life.

Consistent with insufficiency of lipid fuels in supporting neonatal survival, the levels of circulating and hepatic TAG, FFA and β-hydroxybutyrate were all elevated upon fat administration into the LFM-fed pups, even in the presence of gluconeogenesis inhibitors that prevented the rescue of the dying pups (Fig. 5C–H). As expected, circulating and hepatic β-hydroxybutyrate were blocked by FAO inhibitors (Fig. 5E, H). By contrast, fat supplementation in LFM-fed pups increased hepatic glucose production, which was completely blocked by FAO or gluconeogenesis inhibitors (Fig. 5I). The hepatic levels of p-AMPK, as indicators of cellular energy status (38), also correlated with hepatic glucose levels in these treatments (Fig. 5J). As a result, the inhibition of FAO and gluconeogenesis also blocked the increase in blood glucose, brain glucose, and brain ATP production enhanced by fat supplementation (Fig. 5K–M). These data demonstrate that the rescue effect by fat supplementation is dependent on FAO and gluconeogenesis.

### Glucose is a universal energy source for neonates

**Fig. 6:**
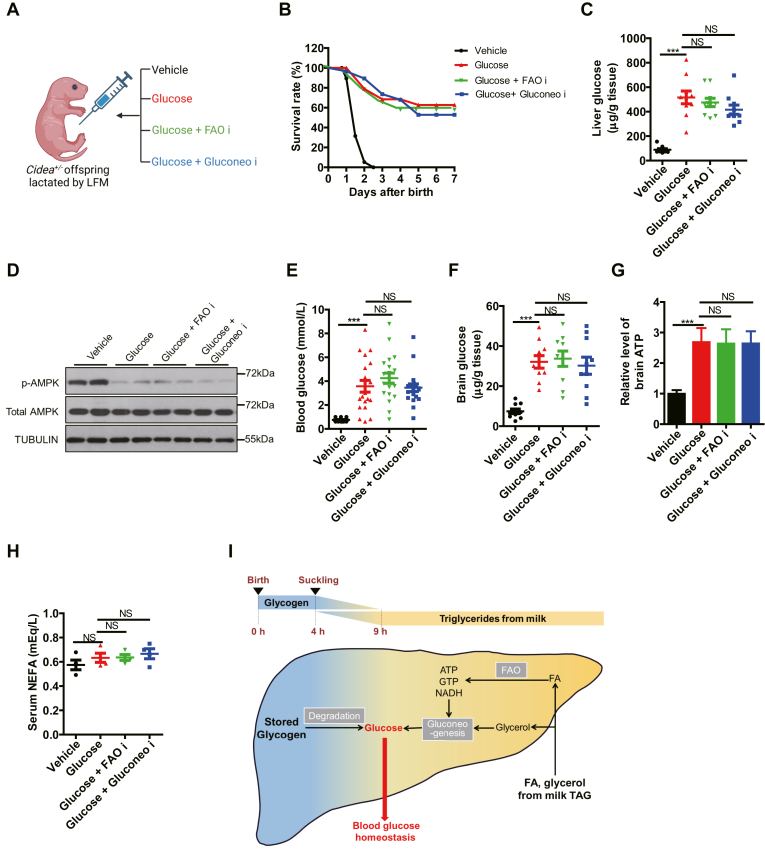
Glucose is a universal energy source for neonates. A: Schematic of the rescue experiments with glucose. From 6 h after birth, the pups lactated by *Cidea*^*−/−*^ females (low-fat milk, LFM) were injected with four solutions every 8 h: vehicle, Glucose, Glucose + FAO i and Glucose + Gluconeo i, respectively. B: The survival rate of different groups in (A) (n = 15). C: Hepatic levels of glucose (n = 10). D: Protein expression in livers by Western blotting (n = 2). E: Blood glucose level (n = 20). F, G: Brain glucose (F) and ATP (G) level (n = 10). H: The level of serum NEFA (n = 4). I: Model depicting the role of glycogen and milk TAG in maintaining neonatal blood glucose homeostasis. Data are presented as mean ± s.e.m. Data were analyzed using a two-tailed Student’s *t* test. ∗*P* < 0.05, ∗∗*P* < 0.01, ∗∗∗*P* < 0.001. FAO, fatty acid oxidation; NEFA, nonesterified fatty acid; TAG, triacylglyceride.

To determine if the limited glucose supply caused by LFM feeding is responsible for the neonatal death, we performed a rescue experiment by intraperitoneal glucose injections into *Cidea*^*+/−*^ pups receiving LFM (50 μl/g of 5% glucose every 8 h after birth until weaning) (Fig. 6A). This glucose supply rescued the perinatal lethality by ∼60% (*P* < 0.001, Fig. 6B). In sharp contrast to the cases of lipid replenishment in Fig. 5, the addition of FAO or gluconeogenesis inhibitors had little impact on the rescue of lethality by glucose (Fig. 6B). Accordingly, glucose injection replenished the depleted glucose levels in the liver, whereas FAO or gluconeogenesis inhibitor failed to prevent the replenishment (Fig. 6C). At the molecular level, phosphorylation of hepatic AMPK was consistently lowered in the glucose-injected neonates, further reflecting the restored energy supply in hepatocytes (Fig. 6D). Similar to the liver, this also occurred to blood and brain glucose (Fig. 6E, F). Similar results were observed in the case of ATP concentration in the brain (Fig. 6G). By contrast, circulating NEFA levels were not affected by glucose injection (Fig. 6H). Taken together, the data suggested that glucose supply bypassed the requirements of FAO and gluconeogenesis in lipid consumption, therefore established a universal role of glucose as the fuel for neonatal survival and the convergence of metabolic pathways for supplying the essential fuel (Fig. 6I).

## Discussion

Glucose is the vital energy fuel for a new life. Therefore, maintaining blood homeostasis for a stable and sufficient supply of the vital fuel is paramount at the moment of birth, when maternal supply ceases. Our study demonstrates that neonatal liver is the center for glucose production and supply, by integrating different energy substrates. Unexpectedly, this funneling into glucose is achieved with shifting the metabolic landscape from glycogenolytic to FAO, with the latter of which supports gluconeogenesis. In early hours after birth, the neonates quickly mobilize stored glycogen for hepatic glucose until milk suckling. While the main energy source is shifted to lipids after milk suckling, hepatic FAO and gluconeogenesis are both induced and chronically activated. Free fatty acids are actively oxidized in liver to produce ATP and NADH to support gluconeogenesis. Consistent with this notion, lack of milk lipids, blockade of FAO or defective gluconeogenesis all cause neonatal death within 2 days. Moreover, the lethality can all be restored by glucose readministration. Therefore, neonatal liver actively takes advantage of different energy sources to produce glucose to support other organs.

Taking advantage of the liver-specific *Pygl* KO mice, our study demonstrates that the mobilization of hepatic glycogen maintains blood glucose in neonates in early hours after birth prior to milk feeding. As previously reported (39, 40), blood glucose drops sharply at birth within 1 h, followed by a rapid rebound to normal physiological range after 3 h postnatal. The transient neonatal hypoglycemia is accompanied by the secretion of glucagon (41), which turns on the glycogenolysis program by PYGL phosphorylation and GDE induction. Human patients bearing *Pygl* mutations are viable, but with growth retardation in 1–3 years old children (42), supporting glycogen mobilization is important but not essential for early life in human.

Our study unexpectedly revealed that, following the glycogen slashing and the start of milk consumption, fatty acids take the central stage to maintain blood glucose homeostasis, through a relay from FAO to gluconeogenesis. Consistent with this notion, blood glucose started to climb to a stable level after milk suckling even in *Pygl* LKO mice. By contrast, the isogenic mating experiments, using the *Cidea*^*−/−*^ mother that provide low-lipid milk (15), demonstrated that sufficient lipid supply was required for neonatal survival and growth. More importantly, the neonatal lethality caused by limited milk lipids could be rescued by exogenous supplementation of either lipid or glucose. It should be noted that, however, blocking either FAO or gluconeogenesis abolished the rescue effects by lipid resupply, but not those by glucose addition. These results therefore supported a model of metabolic funneling, in which hepatic FAO produces essential molecules to support gluconeogenesis and therefore the supply of glucose to extrahepatic tissues. This does not contradict the paradigm that acetyl-CoA molecules derived from FAO cannot be converted to glucose via gluconeogenesis in mammals. Instead, ATP and NADH produced from FAO are required to support the energy costly processes of gluconeogenesis (43), thereby maintaining a stable supply of glucose to fuel extrahepatic tissues in neonates.

Hepatic FAO can also drive the production of ketone body in neonates. Ketogenesis is induced in neonatal liver after suckling, producing ketone bodies as an alternative fuel for brain and heart. A recent study demonstrates that FAO is required to induce ketogenesis in neonatal heart, which is required for heart mitochondrial activity and offspring survival before waning (44). However, loss of the key ketogenic enzyme Hmgcs2 does not cause lethality until one-week postnatal (45). These data suggest an important but not required role of ketones in early life, in contrast to glucose.

The study may be of value for neonatal medicine in humans, especially in cases of premature infants or inborn metabolic diseases with mutations in FAO oxidation and gluconeogenesis. The affected infants often develop severe hypoglycemia after even a short fasting (20), supporting the concept that lipid oxidation supports glucose production in neonatal. Therefore, while lipid supplementation is important for premature infants, direct glucose supply maybe preferred for infants bearing deficiencies in fatty acid transport, FAO or gluconeogenesis. Moreover, the lipid-loaded and glucogenic liver in neonates might appear similar to diabetic liver in adults, who often exhibit increased lipid utilization and glucose production (46). Nevertheless, the tight control of hormonal regulations, including, by insulin and glucagon, ensure the proper function of neonatal liver with minimal risk of developing diabetes despite the high metabolic load. Hence, further investigation of the metabolic coordination and hormonal regulation in neonatal liver may also shed light to liver function and metabolic diseases in adult humans.

In conclusion, we show here that a shift of energy substrate from internal hepatic glycogen stores to external milk lipid, which coincides with drastic nutritional changes at birth, alters hepatic metabolism processes. This metabolism adaptation of neonates to nutritional environment plays a key role in regulating glucose homeostasis, and is also important for postnatal survival and growth.

## Data availability

The authors will supply the relevant data in response to reasonable requests.

## Supplemental data

This article contains supplemental data.

## Conflict of interest

The authors declare that they have no conflict of interest with the contents of this article.

## Supplemental data

Supplemental Figure S1 The absolute levels of serum fuels and protein and mRNA levels of several pathways. A–F: The absolute levels of serum fuels determined by kits (n = 6). G: Key protein expression in neonatal liver by Western blotting (n = 2). H–M: mRNA levels of several pathways related to gluconeogenesis (H), glycolysis (I), FA transport and FAO (J), ketogenesis (K), FA *de novo* synthesis (L) and cholesterol synthesis (M), respectively (n = 6). Data are presented as mean ± s.e.m. Data were analyzed using a two-tailed Student’s *t*-test. ∗*P* < 0.05, ∗∗*P* < 0.01, ∗∗∗*P* < 0.001.

Supplemental Figure S2 The performance of *Pygl*^*−/−*^ mice. A: Pygl is the limited enzyme in glycogen degradation. B: The survival rate (n = 20). C: The body weight (n = 6). D: The protein expression levels of G6PC, PCK1, CPT1A, p-CREB and total CREB (n = 2). Data are presented as mean ± s.e.m. Data were analyzed using a two-tailed Student’s *t*-test. ∗*P* < 0.05, ∗∗*P* < 0.01, ∗∗∗*P* < 0.001.

Supplemental Figure S3 The composition of milk lactated by *Cidea*^+/+^ female and *Cidea*^*−*/*−*^ female, and the lung morphology of their offsprings. A: The TAG level in milk detected by a kit. B: Heatmap of lipidomics (n = 4), metabolomics (n = 5), proteomics (n = 3) of milk from *Cidea*^*+/+*^ and *Cidea*^*−/−*^ females. C: Relative levels of TAG, Diacylglycerol (DAG), free fatty acids (FA), lysophosphatidyl (LPC), choline phosphatidylcholine (PC), phosphatidylethanolamine (PE), phosphatidylinositol (PI), phosphatidylserine (PS), and sphingomyelin (SM) in milk of *Cidea*^*+/+*^ and *Cidea*^*−/−*^ females according to lipidomics. D–F: levels of milk protein (n = 10) (D), lactose (n = 8) (E) and miRNA (n = 6) (F) in *Cidea*^*+/+*^ and *Cidea*^*−/−*^ females, respectively. G: Representative lung morphology (n = 3). Data are presented as mean ± s.e.m. Data were analyzed using a two-tailed Student’s *t*-test. ∗*P* < 0.05, ∗∗*P* < 0.01, ∗∗∗*P* < 0.001.
