# Supplementary figures and images for: Metabolic switch from glycogen to lipid in the liver maintains glucose homeostasis in neonatal mice
Source: J Lipid Res. 2023 Oct 11;64(10):100440. doi: 10.1016/j.jlr.2023.100440 (PMC10568567; doi:10.1016/j.jlr.2023.100440)

Supplementary Fig. 1

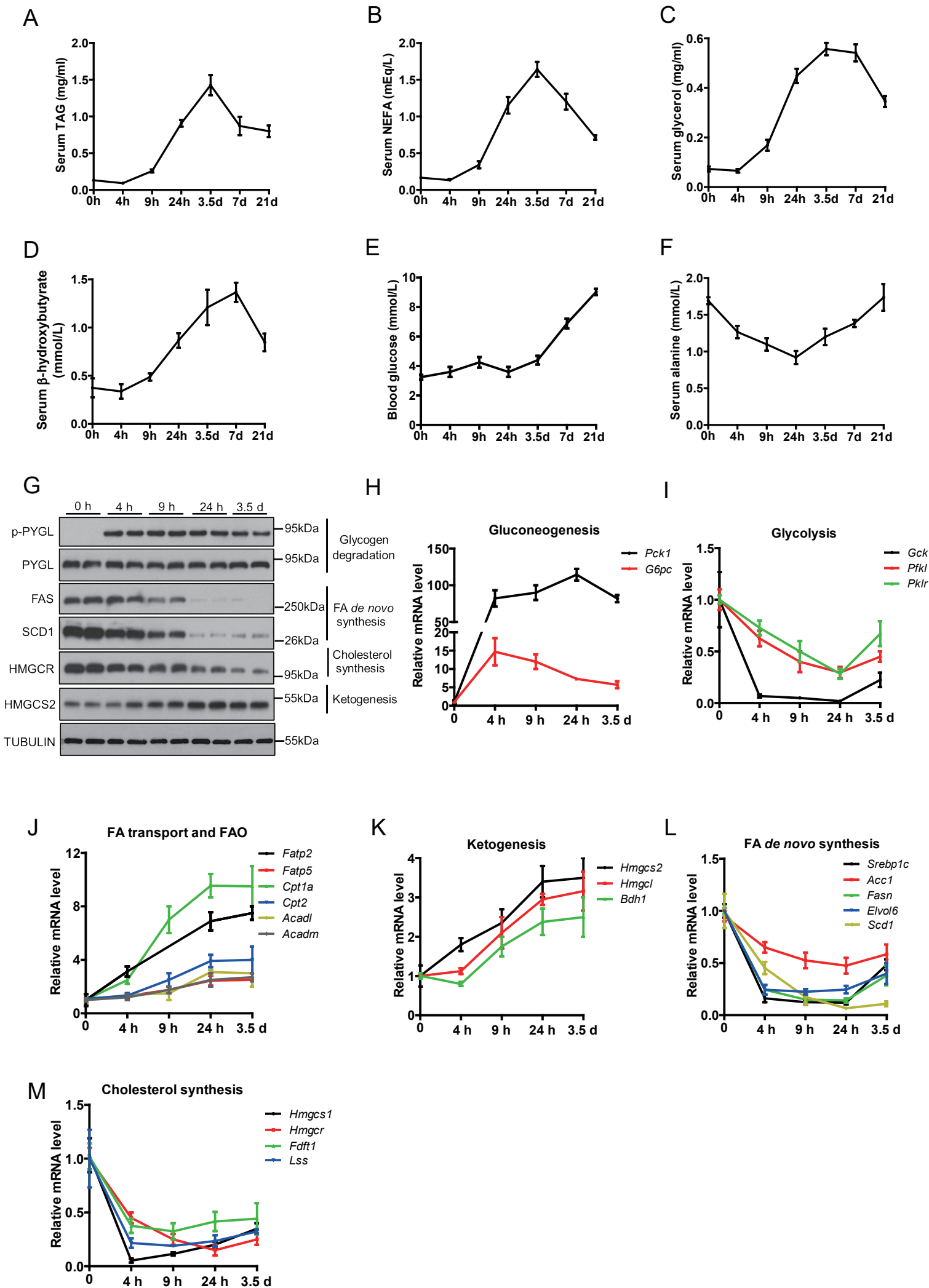

Supplement: Supplemental Figure S1 — The absolute levels of serum fuels and protein and mRNA levels of several pathways. A–F: The absolute levels of serum fuels determined by kits (n = 6). G: Key protein expression in neonatal liver by Western blotting (n = 2). H–M: mRNA levels of several pathways related to gluconeogenesis (H), glycolysis (I), FA transport and FAO (J), ketogenesis (K), FA de novo synthesis (L) and cholesterol synthesis (M), respectively (n = 6). Data are presented as mean ± s.e.m. Data were analyzed using a two-tailed Student’s t-test. ∗P < 0.05, ∗∗P < 0.01, ∗∗∗P < 0.001. [file mmc1.pdf]

## Supplementary Fig. 2

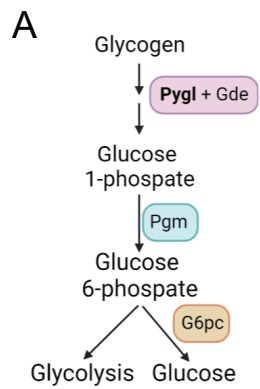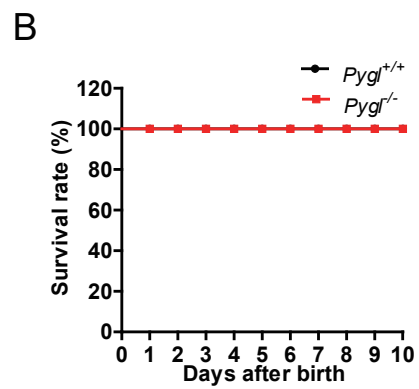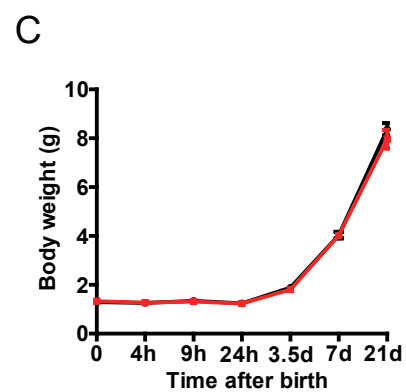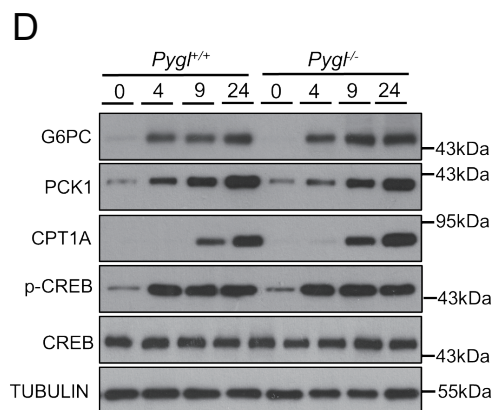

Supplement: Supplemental Figure S2 — The performance of Pygl−/− mice. A: Pygl is the limited enzyme in glycogen degradation. B: The survival rate (n = 20). C: The body weight (n = 6). D: The protein expression levels of G6PC, PCK1, CPT1A, p-CREB and total CREB (n = 2). Data are presented as mean ± s.e.m. Data were analyzed using a two-tailed Student’s t-test. ∗P < 0.05, ∗∗P < 0.01, ∗∗∗P < 0.001. [file mmc2.pdf]

Supplementary Fig. 3

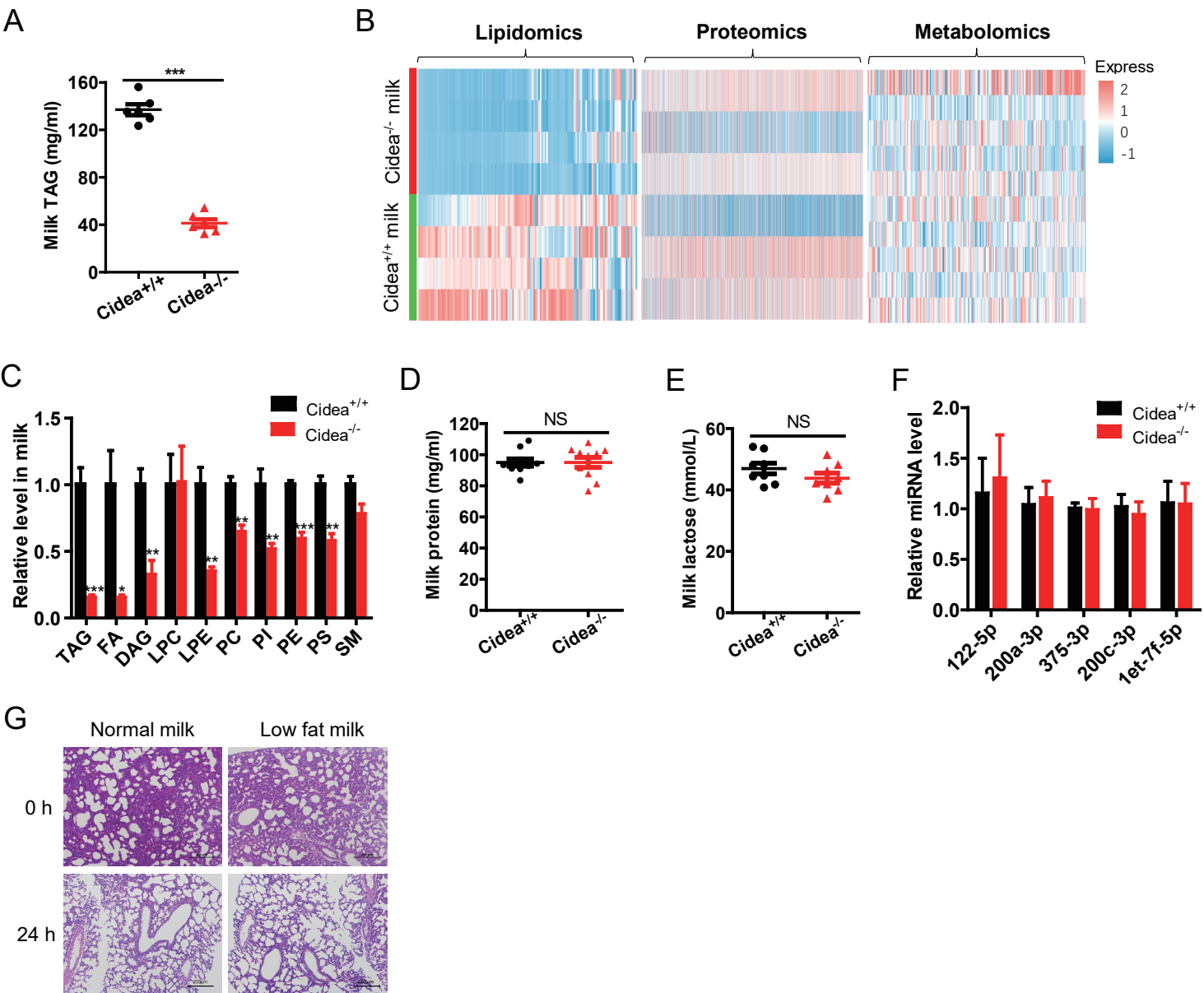

Supplement: Supplemental Figure S3 — The composition of milk lactated by Cidea+/+ female and Cidea−/− female, and the lung morphology of their offsprings. A: The TAG level in milk detected by a kit. B: Heatmap of lipidomics (n = 4), metabolomics (n = 5), proteomics (n = 3) of milk from Cidea+/+ and Cidea−/− females. C: Relative levels of TAG, Diacylglycerol (DAG), free fatty acids (FA), lysophosphatidyl (LPC), choline phosphatidylcholine (PC), phosphatidylethanolamine (PE), phosphatidylinositol (PI), phosphatidylserine (PS), and sphingomyelin (SM) in milk of Cidea+/+ and Cidea−/− females according to lipidomics. D–F: levels of milk protein (n = 10) (D), lactose (n = 8) (E) and miRNA (n = 6) (F) in Cidea+/+ and Cidea−/− females, respectively. G: Representative lung morphology (n = 3). Data are presented as mean ± s.e.m. Data were analyzed using a two-tailed Student’s t-test. ∗P < 0.05, ∗∗P < 0.01, ∗∗∗P < 0.001. [file mmc3.pdf]
