# Supplementary material for: Metabolic switch from glycogen to lipid in the liver maintains glucose homeostasis in neonatal mice
Source: J Lipid Res. 2023 Oct 11;64(10):100440. doi: 10.1016/j.jlr.2023.100440 (PMC10568567; doi:10.1016/j.jlr.2023.100440)
Supplement: Supplemental Table S1 [file mmc4.pdf]

Supplementary Table 1. Sequences of primers used for qRT-PCR analysis.

	Forward (5'-3')	Reverse (5'-3')
Actin	GGCTGTATTCCCCTCCATCG	CCAGTTGGTAACAATGCCATGT
Ppara	ACGGCAATGGCTTTATCA	CGCTGCGTCGGACTCGGT
Fatp2	TCCTCCAAGATGTGCGGTACT	TAGGTGAGCGTCTCGTCTCG
Fatp5	CTACGCTGGCTGCATATAGATG	CCACAAAGGTCTCTGGAGGAT
Cpt1a	ACCACTGGCCGCATGT	CTCCATGGCGTAGTAGTTGCT
Cpt2	CAGCACAGCATCGTACCCA	TCCCAATGCCGTTCTCAAAAT
Cox4	CGGCGTGACTACCCCTTG	TGAGGGATGGGGCCATACA
Cyto C	CCAAATCTCCACGGTCTGTTC	ATCAGGGTATCCTCTCCCCAG
Acadl	GCATCAACATCGCAGAGAAA	ACGCTTGCTCTTCCCAAGTA
Acadm	GCTAGTGGAGCACCAAGGAG	CCAGGCTGCTCTCTGGTAAC
Pck1	GAAGGACAAAGATGGCAAGTT	CGTTTTCCTTAGGGATGTAGC
G6pc	CTGAGCGCGGGCATCATAAT	GATTCTTAGGATCGCCCAGAAAG
Hmgcs2	GAAGAGAGCGATGCAGGAAAC	GTCCACATATTGGGCTGGAAA
Hmgcl	CAGGTGAAGATCGTGGAAGTC	GGAGCCCTGCTTCGGAAAC
Bdh1	ACAAGACACACGCTGTTGTTT	CTCTTCAAGCTGTCCAGTTCC
Gck	GTCTTTTGCAACACTCAGCCA	ATCCGGCTCATCACCTTCTTC
Pfkl	GGAGGCGAGAACATCAAGCC	CGGCCTTCCCTCGTAGTGA
Pklr	TGGCATCGAAAGTGGAAAGC	GATGTGGGACTATGGGAGGG
Srebp1c	GGAGCCATGGATTGCACATT	GCTTCCAGAGAGGAGGCCAG
Acc1	AGCTGATCCTGCGAACCT	GCCAAGCGGATGTAAACT
Fasn	TCCAAGACTGACTCGGCTACTGAC	GCAGCCAGGTTCGGAATGCTATC
Elovl6	AAGCAGTTCAACGAGAACGAA	CGTACAGCGCAGAAAACAGG
Scd1	TTCTTGCGATACACTCTGGTGC	CGGGATTGAATGTTCTTGTCGT
Hmgcs1	AATTGGGCCAAACGCTCCT	TCTCGTACAAGAGAACTGGCTA
Hmgcr	CTGGAATTATGAGTGCCCCAAA	ACGACTGTACTGAAGACAAAGC
Fdft1	ATGGAGTTCGTCAAGTGTCTAGG	CGTGCCGTATGTCCCCATC
Lss	TCGTGGGGGACCCTATAAAAC	CGTCCTCCGCTTGATAATAAGTC
