# Supplementary material for: Metabolic switch from glycogen to lipid in the liver maintains glucose homeostasis in neonatal mice
Source: J Lipid Res. 2023 Oct 11;64(10):100440. doi: 10.1016/j.jlr.2023.100440 (PMC10568567; doi:10.1016/j.jlr.2023.100440)
Supplement: Supplemental Table S2 [file mmc5.pdf]

Supplementary Table 2. Serum chemistry of *Pygl*$^{+/+}$ and *Pygl*$^{-/-}$ mice at 0 h, 4 h, 9 h, 24 h, after birth. (No differences between *Pygl*$^{+/+}$ and *Pygl*$^{-/-}$ mice)

	TAG (mg/ml)		NEFA (mEq/L)		Glycerol (mg/ml)		β-hydroxybutyrate (mmol/L)	
	Pygl$^{+/+}$	*Pygl*$^{-/-}$	*Pygl*$^{+/+}$	*Pygl*$^{-/-}$	*Pygl*$^{+/+}$	*Pygl*$^{-/-}$	*Pygl*$^{+/+}$	*Pygl*$^{-/-}$
0 h	0.13±0.02	0.13±0.01	0.18±0.03	0.19±0.02	0.07±0.02	0.08±0.02	0.38±0.03	0.37±0.03
4 h	0.08±0.02	0.10±0.01	0.14±0.02	0.14±0.02	0.07±0.02	0.05±0.01	0.35±0.05	0.34±0.04
9 h	0.20±0.03	0.16±0.02	0.35±0.07	0.29±0.04	0.21±0.04	0.17±0.03	0.46±0.04	0.50±0.07
24 h	1.05±0.06	0.95±0.05	1.17±0.19	1.00±0.14	0.49±0.06	0.45±.08	0.75±0.07	0.72±0.08
